# Peritonitis

**Published:** 1906-01-13

**Authors:** 


### PERITONITIS.

**The Treatment of Acute Peritonitis** is beset with many difficulties, and is a subject upon which there is a wide divergence of opinion amongst surgeons. Lennander[1] rightly describes the peritoneal cavity as a big lymph space which is everywhere in communication with the lymph vessels and blood-vessels of the subserosa. The absorption through the serosa is quickest and most copious in the centre of the abdomen and through the serosa of the diaphragm, slowest in other parts of the periphery of the abdomen. The absorption ceases or becomes slower in the same proportion as a rich serous or purulent exudation gathers in the abdomen, free or encysted. This is a double advantage to the patient. The toxins are richly diluted, and at the same time their absorption is momentarily prevented or rendered more difficult. The absorption is prevented chiefly by thrombosis and compression of lymph spaces, lymph capillaries and blood capillaries. In every case of more advanced acute peritonitis the cause of intestinal paralysis is a toxic (and microbic) poisoning of the walls of the intestine, with anatomical changes in the lymph spaces, and of the ganglionic and nerve cells of Auerbach's plexus. It is probable that the normal peristalsis of the intestine has its origin in the chemical and mechanical qualities of the intestinal contents which produce a greater or less irritation of the mucosa. This irritation is transmitted to Auerbach's plexus, which furnishes both muscular layers with a co-ordinate innervation. The position of Auerbach's plexus under the longitudinal layer of muscles is near the serosa. If this becomes infected, toxins, microbes, lymphangitis, and other inflammatory changes quickly reach the plexus, where they produce greater or lesser changes, which at worst effect the death of the plexus. The affected part of the intestine will probably never again be capable of movement. Next to the absorption in masses of toxins and microbes at the onset of a peritoneal infection, there is nothing more dangerous during the development of a peritonitis than the occurrence of intestinal paralysis. The walls of the paralysed gut become permeable to toxins, and soon even to microbes, so that they are not only admitted into the lymphatic vessels and veins of the intestine in increased quantity, and are carried off to the thoracic duct and to the liver, but toxins and microbes pass straight through the wall of the intestine into the general peritoneal cavity. Both infections spread with tremendous speed over the large surface offered by the serosa of the small intestine and the mesentery. The wall of the intestine is now attacked both from the serosa and the mucosa, and the organism is poisoned both from the peritoneal cavity and from the lumen of the intestine. From this it is evident that an acute peritonitis in the centre of the abdomen is much more dangerous than one in its periphery. It is also clear that a patient with acute peritonitis should be operated upon as soon as circumstances permit before the infection has had time to spread much and before it has time to cause paralysis of the intestine, with the attendant evil effects to which reference has been made. The objects of an operation for acute peritonitis are:—(1) To remove liquid exudation; (2) to remove or treat the source of the infection through extirpation, resection, sutures, plastic operations, tamponing, drainage; (3) to clean the infected portions of the peritoneal cavity; (4) to empty and drain the intestine when it is paralysed; (5) to provide for further discharge, by means of drainage, if possible, or at least to separate, by tamponing and draining, the portions of the serosa most affected from other portions of the peritoneal cavity, which, as a rule, is easily carried out at the periphery of the abdomen, but scarcely in the middle of it. Lennander does not use gauze for tamponing, but a very thick sort of cotton yarn, such as is used for wicks. Amongst the yarn is placed one or two india-rubber tubes. In all places where the yarn would touch the parietal peritoneum, one has to cover it by very thin india-rubber tissue. By doing so one is enabled to remove the yarn without causing pain. The day after the operation one begins to instil peroxide of hydrogen or glycerin with peroxide of hydrogen through the tubes. After two days or so one may remove one or both tubes and pull out the nearest threads. There is no difficulty in draining the pelvis through the vagina, or, in the case of male patients, through the ischio-rectal fossa. Nor is there any difficulty in draining the right subphrenic space by means of a lumbar incision. The position of the patient after the operation has to be chosen chiefly with respect to the drainage. In cases of spreading peritonitis the most suitable position is a nearly sitting one, with the foot-end of the bed more or less elevated. At every operation for spreading peritonitis an assistant must be prepared to introduce saline solution into a vein. If a small pulse does not improve, as soon as the abdominal incision is made the infusion into the vein should be administered. Lennander always irrigates the peritoneal cavity with saline solution in cases of universal or widespread purulent peritonitis where the presence of a copious liquid exudation manifestly shows that the absorbing power of the peritoneum is little or none; but not before he has removed the source of infection, if this has been possible. In other cases he tries to wipe up all pus and contents of the intestine as may have escaped by means of compresses wrung out in saline solution. It is necessary to irrigate if one has been obliged to extrude the small intestine. If experiments show that the washing of the peritoneal cavity with saline solution always produces a considerably increased leucocytosis one will not have to fear so much in future, as now, when treating a purulent peritonitis, a washing of surrounding sound regions of the peritoneal cavity. On the contrary, after the diseased cavity has been previously cleansed as well as possible, such a washing may be considered as a preventive against the spreading of the peritonitis by, for instance, acute lymphangitis of the serosa. Where the intestines are paralysed and loaded with toxic products an opening must be made in the small gut in order to empty it of its contents. This opening may then be closed, or kept open for drainage and closed at a later date. Van Buren Knott [2] practically adopts the same method of drainage. The incision is made in the median line above the umbilicus when the source of infection is due to contamination from the upper abdominal viscera. The diseased parts are repaired, and a two-inch incision is then made in the median line just above the symphysis pubis and through this a large rubber tube, one to one and a half inches in diameter, split from end to end and carrying a gauze wick, is introduced to the bottom of the pelvis and into the vagina in the case of a female. The abdominal cavity is now thoroughly washed out with gallons of hot saline solution, and the upper incision closed. Before tying the last stitch, enough of the saline solution is introduced through a funnel to entirely fill the abdomen. The lower wound is left open, and the patient raised into a sitting posture. This posture is maintained in the bed, the head of which has been raised from 24 to 30 inches from the floor. For infections originating in the lower abdomen or pelvis, the incision is made below the umbilicus, extending to the symphysis. The technique is the same. Knott claims that by adopting this method the mortality of this disease has been reduced from 90 per cent. to 11 per cent. approximately.

[1] Edinburgh Med. Jour., August, 1905, p. 105. [2] Annals of Surg., July, 1905, p. 75.

